# Global research priorities for interpersonal violence prevention: a modified Delphi study

**DOI:** 10.2471/BLT.16.172965

**Published:** 2016-10-20

**Authors:** Christopher R Mikton, Masako Tanaka, Mark Tomlinson, David L Streiner, Lil Tonmyr, Bandy X Lee, Jane Fisher, Kathy Hegadoren, Joam Evans Pim, Shr-Jie Sharlenna Wang, Harriet L MacMillan

**Affiliations:** a Department of Health and Social Sciences, University of the West of England, Bristol Frenchay Campus, Coldharbour Lane, Bristol, BS16 1QY, England.; b McMaster University, Hamilton, Canada.; c Stellenbosch University, Stellenbosch, South Africa.; d Public Health Agency of Canada, Ottawa, Canada.; e Yale University, New Haven, United States of America (USA).; f Monash University, Melbourne, Australia.; g University of Alberta, Edmonton, Canada.; h Center for Global Nonkilling, Honolulu, USA.; i Danish Institute against Torture, Copenhagen, Denmark.

## Abstract

**Objective:**

To establish global research priorities for interpersonal violence prevention using a systematic approach.

**Methods:**

Research priorities were identified in a three-round process involving two surveys. In round 1, 95 global experts in violence prevention proposed research questions to be ranked in round 2. Questions were collated and organized according to the four-step public health approach to violence prevention. In round 2, 280 international experts ranked the importance of research in the four steps, and the various substeps, of the public health approach. In round 3, 131 international experts ranked the importance of detailed research questions on the public health step awarded the highest priority in round 2.

**Findings:**

In round 2, “developing, implementing and evaluating interventions” was the step of the public health approach awarded the highest priority for four of the six types of violence considered (i.e. child maltreatment, intimate partner violence, armed violence and sexual violence) but not for youth violence or elder abuse. In contrast, “scaling up interventions and evaluating their cost–effectiveness” was ranked lowest for all types of violence. In round 3, research into “developing, implementing and evaluating interventions” that addressed parenting or laws to regulate the use of firearms was awarded the highest priority. The key limitations of the study were response and attrition rates among survey respondents. However, these rates were in line with similar priority-setting exercises.

**Conclusion:**

These findings suggest it is premature to scale up violence prevention interventions. Developing and evaluating smaller-scale interventions should be the funding priority.

## Résumé

**Objectif:**

Définir les priorités mondiales de recherche pour la prévention de la violence interpersonnelle à l'aide d'une approche systématique.

**Méthodes:**

Les priorités de recherche ont été établies au cours d'un processus en trois cycles, comprenant deux questionnaires. Durant le premier cycle, 95 experts mondiaux dans le domaine de la prévention de la violence ont proposé des questions de recherche, qui seraient classées au cours du deuxième cycle. Ces questions ont été rassemblées et organisées suivant l'approche en quatre étapes de la prévention de la violence axée sur la santé publique. Lors du deuxième cycle du processus, 280 experts internationaux ont classé par ordre d'importance les recherches concernant les quatre étapes et les différentes sous-étapes de cette approche axée sur la santé publique. Au cours du troisième cycle, 131 experts internationaux ont classé par ordre d'importance des questions de recherche détaillées sur l'étape considérée comme prioritaire au cycle n°2.

**Résultats:**

Lors du deuxième cycle, « élaborer, mettre en œuvre et évaluer les interventions » était l'étape de l'approche de santé publique jugée prioritaire pour quatre des six types de violence considérés (à savoir maltraitance de l'enfant, violence à l'encontre du partenaire intime, violence armée et violence sexuelle) mais non pour la violence chez les jeunes ou la maltraitance des personnes âgées. À l'inverse, « étendre les interventions et évaluer leur rapport coût-efficacité » était jugé comme l'étape la moins importante pour tous les types de violence. Lors du troisième cycle, « élaborer, mettre en œuvre et évaluer les interventions » relatives au rôle des parents ou aux lois règlementant l'utilisation des armes à feu était considéré comme le domaine de recherche prioritaire. Les principales limitations de l'étude étaient les taux de réponse et d'abandon de l'étude par les répondants. Ces taux étaient toutefois comparables à ceux d'exercices similaires de définition de priorités.

**Conclusion:**

Ces résultats laissent entendre qu'il est prématuré d'étendre les interventions de prévention de la violence. La priorité de financement devrait porter sur l'élaboration et l'évaluation d'interventions de moindre ampleur.

## Resumen

**Objetivo:**

Establecer prioridades de investigación globales para la prevención de la violencia interpersonal mediante el uso de un enfoque sistemático.

**Métodos:**

Se identificaron las prioridades de investigación en un proceso de tres etapas que incluía dos encuestas. En la primera etapa, 95 expertos mundiales en la prevención de la violencia propusieron preguntas de investigación para su clasificación en la segunda etapa. Las preguntas se recopilaron y organizaron según el enfoque de salud pública de cuatro fases sobre la prevención de la violencia. En la segunda etapa, 280 expertos internacionales clasificaron la importancia de la investigación en cuatro fases, y en distintas subfases, del enfoque de salud pública. En la tercera fase, 131 expertos internacionales clasificaron la importancia de las preguntas de investigación detalladas sobre la fase de salud pública que, en la segunda fase, se consideraron de mayor prioridad.

**Resultados:**

En la segunda etapa, la fase de “desarrollo, implementación y evaluación de intervenciones” fue la que se consideró de mayor prioridad del enfoque de salud pública para cuatro de los seis tipos de violencia considerados (esto es, maltrato infantil, violencia conyugal, violencia armada y violencia sexual), pero no para la violencia juvenil o el abuso de ancianos. Sin embargo, la fase de “incremento de las intervenciones y evaluación de su rentabilidad” obtuvo la menor calificación de todos los tipos de violencia. En la tercera etapa, la investigación sobre la fase de “desarrollo, implementación y evaluación de intervenciones” que abordó la paternidad o las leyes para regular el uso de las armas de fuego se consideró la de mayor prioridad. Las limitaciones fundamentales del estudio fueron las tasas de respuesta y abandono entre los encuestados. No obstante, dichas tasas concordaron con los ejercicios de prioridad similar.

**Conclusión:**

Los resultados sugieren que aún es pronto para ampliar las intervenciones para prevenir la violencia. La mayor prioridad debería ser el desarrollo y la evaluación de intervenciones a menor escala.

## ملخص

**الهدف:**

تحديد الأولويات البحثية العالمية لمنع العنف بين الأشخاص باستخدام نهج منظم.

**الطريقة:**

تم تحديد الأولويات البحثية من خلال عملية تتألف من ثلاث مراحل تتضمن دراستين. اقترح 95 خبيرًا عالميًا في منع العنف في المرحلة الأولى بعض الأسئلة البحثية ليتم النظر فيها في المرحلة الثانية. وقد تم ترتيب الأسئلة وتنظيمها وفقًا لنهج الصحة العامة المكوّن من أربع خطوات لمنع العنف. ثم قام 280 خبيرًا دوليًا في المرحلة الثانية بترتيب أهمية البحث في أربع خطوات، والخطوات الفرعية العديدة التي يتألف منها مجال الصحة العامة. أما في المرحلة الثالثة، فقد شهدت قيام 131 خبيرًا دوليًا بترتيب أهمية الأسئلة البحثية المفصلة في الخطوة المتعلقة بالصحة العامة التي تم منحها الأولوية القصوى في المرحلة الثانية.

**النتائج:**

كان "تطوير عمليات التدخل وتنفيذها وتقييمها" في المرحلة الثانية هو الخطوة المتعلقة بمجال الصحة العامة التي حازت على الأولوية القصوى لأربعة أنواع من العنف من أصل ستة (وهي المتمثلة في سوء معاملة الأطفال، وعنف الشريك الحميم، والعنف المسلح، والعنف الجنسي)، لكنها لم تنل نفس درجة الأولوية لفئات العنف لدى الشباب، أو إساءة معاملة كبار السن. في المقابل، تم تصنيف "زيادة عمليات التدخل وتقييم فعاليتها من حيث التكلفة" كأدنى مرتبة من بين جميع أنواع العنف. وقد تم منح البحث في "تطوير عمليات التدخل وتنفيذها وتقييمها" – الذي يتناول موضوع تربية الأبناء أو القوانين المنظمة لاستخدام الأسلحة النارية – الأولوية القصوى في المرحلة الثالثة. وكانت القيود الرئيسية للدراسة تتمثل في معدلات الاستجابة بين المشاركين في الاستطلاع، ومع ذلك، كانت هذه المعدلات متماشية مع عمليات مماثلة لتحديد الأولويات.

**الاستنتاج:**

تشير هذه النتائج إلى أنه من السابق لأوانه تصعيد عمليات التدخل لمنع العنف، حيث ينبغي أن يتم توجيه الأولوية في التمويل إلى تطوير عمليات التدخل على نطاق أقل وتقييم تلك العمليات.

## 摘要

**目的:**

旨在运用系统的方法确定人际暴力预防的全球研究重点。

**方法:**

我们通过三轮过程确定了研究重点。这三轮过程中包含两项调查。 在第 1 轮，全球 95 名暴力预防领域的专家提出了将在第 2 轮中排序的调查问题。我们根据针对暴力预防的四步骤公共卫生方法对问题进行了核对和整理。 在第 2 轮，280 名国际专家通过公共卫生方法的四个步骤以及多种子步骤对调查的重要性进行了排序。 在第 3 轮，131 名国际专家根据公共卫生步骤对详细调查问题的顺序进行了排序，确定出第 2 轮中的最高优先级。

**结果:**

在第 2 轮中，“制定、实施和评估预防措施”是公共卫生方法中确定六种暴力类型中四种暴力类型（即虐童、家暴、武装暴力和性暴力）优先级最高的步骤，但不适用于青少年暴力或老人虐待。 与之相反，针对所有暴力类型，“扩大干预及评估其成本效益”的排序最低。 在第 3 轮，针对解决子女教养问题以及轻武器使用法律的“制定、实施和评估干预措施”被列为最高优先级。 本调查的主要限制为调查受访者的响应和流失率。 但是，这些比率与类似的优先级设定练习一致。

**结论:**

调查结果显示扩大暴力预防干预为时尚早。 应优先为制定和评估较小范围的干预提供资金资助。

## Резюме

**Цель:**

Определить приоритеты глобальных исследований для предотвращения межличностного насилия, используя систематический подход.

**Методы:**

Приоритеты исследований были определены в ходе состоящего из трех раундов процесса, в рамках которого было проведено два опроса. В ходе первого раунда 95 специалистов по предупреждению насилия со всего мира предложили предметы исследований, которые предстояло расположить в порядке приоритетности в ходе второго раунда. Вопросы были систематизированы и организованы в соответствии с четырехэтапным подходом к предупреждению насилия с позиций общественного здравоохранения. В ходе второго раунда 280 международных экспертов дали оценку важности исследования в четырех этапах и различных подэтапах подхода с позиций общественного здравоохранения. В ходе третьего раунда международные эксперты в количестве 131 человека дали оценку важности конкретных предметов исследования на этом этапе подхода с позиций общественного здравоохранения, которому был присвоен наивысший приоритет во втором раунде.

**Результаты:**

Во втором раунде наивысший приоритет для четырех из шести типов насилия (жестокое обращение с детьми, насилие со стороны интимного партнера, вооруженное насилие и сексуальное насилие, но не случаи насилия среди молодежи или жестокого обращения с пожилыми людьми) был присвоен этапу подхода с позиций общественного здравоохранения «разработка, осуществление и оценка вмешательств». Для сравнения: «наращивание вмешательств и оценка их экономической эффективности» получили наименьший приоритет для всех типов насилия. В третьем раунде наивысший приоритет был присвоен исследованиям «разработки, осуществления и оценки вмешательств», относящихся к выполнению родительских обязательств или законам, регулирующим использование огнестрельного оружия. Главные недостатки исследования были связаны с долями ответивших и выбывших среди участвующих в опросе. Однако размер этих долей не выходил за рамки, характерные для аналогичных работ по определению приоритетов.

**Вывод:**

На основании результатов можно говорить о том, что в настоящий момент проводить наращивание вмешательств, нацеленных на предупреждение насилия, преждевременно. Разработка и оценка менее масштабных вмешательств должны стать приоритетным направлением финансирования.

## Introduction

Almost half a million people are victims of homicide every year^1^ and one in three women has experienced violence from an intimate partner at some point during her life.^2^ Furthermore, a quarter of adults report having been physically abused in childhood and one in five women and one in 13 men report having been sexually abused in childhood.^3^^,^^4^ Interpersonal violence during childhood can have serious, lifelong consequences that affect mental and physical health, academic and job performance and social functioning.^5^^,^^6^ In addition, interpersonal violence, which includes child maltreatment, intimate partner violence, youth violence, armed violence, sexual violence and elder abuse (Box 1), create an economic burden on society.^7^

Box 1 **Main types of violence, survey of global research priorities for violence prevention, 2010–2013**

**Child maltreatment**

The abuse or neglect of a child younger than 18 years. It includes all types of physical and emotional ill treatment, sexual abuse, neglect, negligence and commercial or other exploitation that result in actual or potential harm to the child’s health, survival, development or dignity in the context of a relationship of responsibility, trust or power. Exposure to intimate partner violence is sometimes included as a form of child maltreatment.

**Intimate partner violence**

Behaviour by an intimate partner or ex-partner that causes physical, sexual or psychological harm. It includes physical aggression, sexual coercion, psychological abuse and controlling behaviours.

**Youth violence**

Violence occurring between people aged 10 to 29 years of age. It includes all types of physical and emotional ill treatment and generally takes place outside of the home. It also includes harmful behaviours that may start early and continue into adulthood. Some violent acts, such as assault, can lead to serious injury or death; others, such as bullying, slapping or hitting, may result more in emotional than physical harm.

**Armed violence**

The intentional use of physical force, threatened or actual, with arms against another person or group that results in loss, injury, death or psychosocial harm to an individual or individuals and that can undermine a community's development, achievements and prospects.

**Sexual violence**

Any sexual act or attempt to obtain a sexual act – including unwanted sexual comments or advances or acts to traffic a person for sexual exploitation – directed against a person using coercion by any person regardless of their relationship to the victim, in any setting, including but not limited to the home and work. It also includes rape, which is defined as physically forced or otherwise coerced penetration, however slight, of the vulva or anus using a penis, another body part or an object.

**Elder abuse**

A single or repeated act or lack of appropriate action that occurs within any relationship where there is an expectation of trust and which causes harm or distress to an older person. Elder abuse includes: (i) physical, sexual, psychological, emotional, financial and material abuse; (ii) abandonment; (iii) neglect; and (iv) serious loss of dignity and respect.

Note: We provided these definitions to survey respondents.

Over the last two decades, the prevention of interpersonal violence has risen on the international public health agenda.^8^ In May 2016, the World Health Organization (WHO) adopted a global plan of action to strengthen the role of health systems in addressing interpersonal violence, particularly against women and girls and against children. The 17 sustainable development goals (SDGs) recently adopted by the United Nations include four targets on interpersonal violence: (i) to eliminate all forms of violence against women and girls (target 5.2); (ii) to eliminate all harmful practices against women and girls (target 5.3); (iii) to reduce significantly all forms of violence and related deaths everywhere (target 16.1); and (iv) to end abuse, exploitation, trafficking and all forms of violence against children (target 16.2).^9^

In spite of progress in the past 20 years, major gaps in violence prevention remain. The *Global status report on violence prevention*^1^ reveals that civil and vital registration data on homicide are lacking in 40% of countries. Moreover, fewer than half of all countries have reported conducting population-based surveys on most forms of nonfatal violence, such as child maltreatment, youth violence and elder abuse.^1^ Only 9.3% of all outcome evaluation studies in violence prevention have been conducted in low- and middle-income countries and there is no indication that this is increasing, despite over 85% of violent deaths occurring in these countries.^10^

Research has a major role to play in reducing the global burden of interpersonal violence, by: (i) clearly defining the magnitude and distribution of violence; (ii) identifying risk and protective factors; (iii) developing effective interventions that target these factors to prevent and respond to violence; and (iv) increasing understanding of the legislative and policy environment and the human, institutional and financial resources required to scale up effective interventions. However, current research remains under-resourced relative to the burden of the problem, it is fragmented and disproportionately focused on high-income countries. 

A systematic and transparent process of establishing global research priorities can provide useful guidance on allocating scarce resources more equitably and on developing a coherent research agenda.^11^^,^^12^ Priority-setting exercises on research have long been carried out in other health fields.^12^^–^^15^ However, in the field of interpersonal violence, such an exercise has only been conducted on child maltreatment and intimate partner violence in high-income countries.^16^

**Fig. 1:**
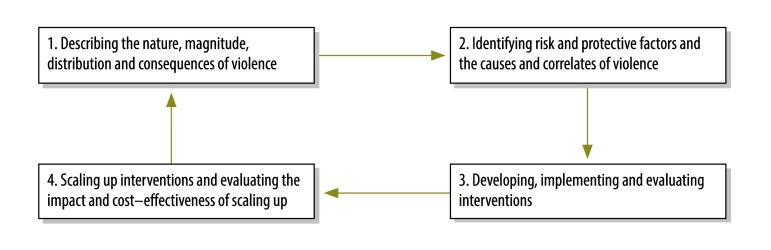
The four steps of the public health approach to violence prevention

Source: Adapted from *The public health approach.*^17^

The aim of this study was to identify global research priorities for the prevention of the main forms of interpersonal violence. The specific objectives were: (i) to rank the priority of the four steps (presented in Fig. 1) of the public health approach to violence prevention for each type of violence (i.e. child maltreatment, intimate partner violence, youth violence, armed violence, sexual violence and elder abuse) and the priority of broad subtypes of research questions within each step;^17^ and (ii) to identify more detailed research priorities for the most highly ranked step. We chose the public health approach because it has been adopted by WHO and other national and global public health agencies to address a broad range of health issues, including violence and unintentional injury, and because it has been gaining prominence outside public health as a way of addressing violence.

## Methods

We carried out the study from October 2010 until September 2013, in consultation with the 20-member Research Agenda Project Group of the WHO-led Violence Prevention Alliance. Our approach combined elements of the Delphi method and the Child Health and Nutrition Research Initiative priority-setting method. The Delphi method is a formal way of developing a consensus that is used when evidence in an area is limited or contradictory. Its aim is to determine, by means of an iterative process, the extent of agreement in that area.^14^ This method often uses a large group of experts to generate research questions. The Child Health and Nutrition Research Initiative priority-setting method is a structured and transparent method that uses predetermined criteria to generate and score research questions systematically. This method assigns a quantitative research priority score to each item on a list of systematically generated research options based on scores given by experts using several criteria. Both methods have been extensively used to establish priorities in health research.^12^^–^^16^^,^^18^

### Study process

**Fig. 2:**
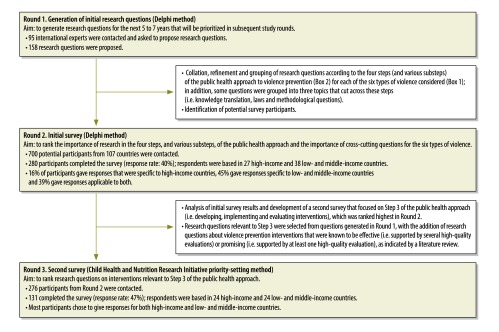
Flow diagram on the study of global research priorities for violence prevention, 2010–2013

The study involved three rounds of expert consultations, which were conducted electronically (Fig. 2). We asked participants to specify their areas of expertise on different forms of violence and, in each round, to give responses related to high-income countries and low- and middle-income countries, respectively. Respondents could give the same ranking to more than one research item if they judged them of equal priority. Given the broad scope of this exercise, which was the prevention of all the main forms of interpersonal violence globally, we regarded the Delphi method as an ideal way of asking a large group with extensive expertise to generate initial research questions (round 1) and to rank these questions on a scale of 1 to 7, with 1 being the highest priority, as appropriate (round 2). In round 3, the Child Health and Nutrition Research Initiative method was used to identify detailed research priorities for the step of the public health approach that ranked highest in round 2. This method produces a finely graded ranking by scoring each research question on several criteria.^12^ For round 3, survey respondents were asked to grade 34 more detailed intervention research questions by rating them from 1 (“strongly disagree”) to 5 “strongly agree”) along five criteria (Box 2), which we developed from previous priority-setting exercises that used this method,^12^^,^^15^ adapting them slightly. For each of the 34 questions, we calculated the mean rating and expressed it as a percentage (rather than out of 5). For example, if the mean rating across the five criteria was 3.8, we reported 76% (3.8/5).

Box 2 **Criteria for rating research questions on violence prevention, survey of global research priorities, 2010–2013**

1. Significance: this research is important and needs to be carried out in the coming 5 years.

2. Feasibility: it is feasible to design and implement a study that addresses this research question in the coming 5 years.

3. Applicability, including effectiveness: conducting research into this question will influence practice and policy in the coming 5 years.

4. Equity: conducting research into this question will help under-resourced populations in the coming 5 years.

5. Ethics: research into this question can be carried out in an ethical manner in the coming 5 years.

To assess agreement across participants, we calculated intra-class correlations for each item measured on a 5-point scale. Correlations ranged from 0 to 1, with a value of 0.75 or above considered excellent. Respondents were also asked whether it was possible to rank the priority of research according to the type of violence and, if yes, to do so.

Potential respondents were first identified through the extensive global network of collaborators in WHO’s Prevention of Violence Unit and the Violence Prevention Alliance, which includes some 65 organizations internationally. In addition, we asked potential respondents to suggest other experts in their region or country. In the two surveys used in rounds 2 and 3, we provided the definitions of key terms to survey respondents. These surveys were created using the web-based free open source software LimeSurvey^19^ and statistical analyses were carried out using SPSS version 22 (SPSS Inc., Chicago, United States of America) and SAS version 9.3 (SAS Institute, Cary, USA). We obtained ethical approval from McMaster University, Hamilton, Canada.

## Results

### Generation of research questions

The demographic characteristics of the 95 study participants are shown in Table 1 (more detailed information on their region of residence is available from the corresponding author). The participants put forward 158 research items in the expert consultation. The suggested research questions were similar across country income levels. Most experts from low- and middle-income countries suggested research topics specific to these countries, whereas experts from high-income countries proposed topics for both high-income and low- and middle-income countries, either separately or without specifying the country income level. After collation and refinement, we grouped 26 research items according to the four steps (Fig. 1) – and the substeps – of the public health approach to violence prevention. In addition, we grouped 20 items into three topics that cut across these steps (i.e. knowledge translation, laws and methodological questions).

**Table 1:** Study respondents, global research priorities for violence prevention, 2010–2013

Respondent’s characteristic	Study round^a^		
	Round 1	Round 2	Round 3
**No. of respondents**	95	280	131
**Sex of respondents (%)**			
Male	66	56	54
Female	34	44	46
**Country of residence of respondents, (%)**			
High-income country	76	63	61
Low- or middle-income country	24	37	39
**No. of countries or territories represented by respondents**	31	65	48
**No. of countries or territories in WHO region represented by respondents**			
African Region	5	15	8
Region of the Americas	8	12	8
South-East Asia Region	1	3	2
European Region	10	24	20
Eastern Mediterranean Region	4	4	3
Western Pacific Region	3	7	7
**No. of respondents in work setting**			
Academic institution	ND	49	43
Nongovernmental organization	ND	30	28
Research institute	ND	17	23
Government department or agency	ND	15	18
Health-care organization	ND	9	12
Social or community service agency	ND	5	3
Advocacy	ND	0	7
Other	ND	12	0
**No. of respondents with expertise in area^b^**			
Child maltreatment	ND	45	51
Intimate partner violence	ND	40	44
Youth violence	ND	53	51
Armed violence	ND	41	34
Sexual violence	ND	40	42
Elder abuse	ND	16	21
**Proportion of respondents who gave survey responses on specific types of country, (%)**			
On high-income countries only	ND	16^c^	15^d^
On low- and middle-income countries only	ND	45^e^	2^f^
On both types combined	ND	39^g^	0
On both types separately	ND	0	83^h^
**Proportion of respondents who gave survey responses on specific types of violence, (%)^i^**			
Child maltreatment	ND	46	NA
Intimate partner violence	ND	41	NA
Youth violence	ND	58	NA
Armed violence	ND	51	NA
Sexual violence	ND	39	NA
Elder abuse	ND	18	NA

NA: not applicable; ND: not determined; WHO: World Health Organization.

^a^ Descriptions of the three study rounds are given in the main text.

^b^ Around 85% of respondents provided this information in rounds 2 and 3.

^c^ All respondents were from high-income countries.

^d^ Around 84% of respondents were from low- and middle-income countries and 16% were from high-income countries.

^e^ Around 67% of respondents were from low- and middle-income countries and 33% were from high-income countries.

^f^ All respondents were from high-income countries.

^g^ Around 18% of respondents were from low- and middle-income countries and 82% were from high-income countries.

^h^ Around 32% of respondents were from low- and middle-income countries and 68% were from high-income countries.

^i^ The proportion of respondents who gave survey responses on a specific type of violence and also worked with that type of violence was 81% for child maltreatment, 80% for intimate partner violence, 80% for youth violence, 70% for armed violence, 76% for sexual violence and 67% for elder abuse.

### Initial survey

The results of the research question ranking in round 2 are shown in Table 2 for all country income levels combined. There were clear trends in the ranking of the four steps of the public health approach. Step 3 (i.e. developing, implementing and evaluating interventions) was ranked highest for child maltreatment, intimate partner violence, armed violence and sexual violence, whereas step 2 (i.e. identifying risk and protective factors and the causes and correlates of violence) was ranked highest for youth violence and step 1 (i.e. describing the nature, magnitude, distribution and consequences of violence) was highest for elder abuse. Step 4 (i.e. scaling up interventions and evaluating the impact and cost–effectiveness of scaling up) was consistently awarded the lowest priority across all types of violence.

**Table 2:** Rank of research question, by type of violence, survey of global research priorities, 2010–2013

Research question	Rank of research question^a^					
	Child maltreatment (*n* = 127)	Intimate partner violence (*n* = 112)	Youth violence (*n* = 158)	Armed violence (*n* = 141)	Sexual violence (*n* = 105)	Elder abuse (*n* = 51)
**The four steps of the public health approach**						
1. Describing the nature, magnitude, distribution and consequences of violence	3	3	3	2	3	1
2. Identifying risk and protective factors and the causes and correlates of violence	2	2	1	3	2	2
3. Developing, implementing and evaluating interventions	1	1	2	1	1	3
4. Scaling up interventions and evaluating the impact and cost–effectiveness of scaling up	4	4	4	4	4	4
**Step 1 of the public health approach**						
1. Defining and measuring violence	2	4	2	3	3	2
2. Research on the magnitude and distribution of violence	1	1	1	2	1	1
3. Research on the consequences of violence	3	2	3	1	2	3
4. Research on the cost of violence	4	3	4	4	4	4
5. Research on the validity of administrative data	5	NA	NA	NA	NA	NA
**Step 2 of the public health approach**						
1. Research on risk factors	2	2	2	1	2	1
2. Research on protective factors	1	1	1	2	1	2
3. Research on the relationship between collective violence and interpersonal violence	NA	NA	3	NA	NA	NA
**Step 3 of the public health approach**						
1. Evaluating the effectiveness of programmes that target actual violence	1	1	1	1	2	1
2. Evaluating the effectiveness of promising programmes (e.g. targeting risk factors)	3	4	4	4	4	4
3. Evaluating violence prevention policies	4	3	3	2	5	3
4. Developing primary prevention programmes based on country-specific risk factors	2	2	2	3	3	2
5. Identifying subgroups within intervention populations	5	6	5	5	6	5
6. Developing operational programme manuals	6	7	6	6	7	6
7. Developing and evaluating approaches that help individuals in abusive relationships	NA	5	NA	NA	NA	NA
8. Determining prevention approaches for younger age groups	NA	NA	NA	NA	1	NA
**Step 4 of the public health approach**						
1. Research on scaling up programmes that have been shown to be effective	2	2	1	1	3	2
2. Research on the feasibility and acceptability of programmes	3	3	3	3	2	3
3. Research on adapting effective programmes to new contexts	1	1	2	2	1	1
4. Economic analysis, including cost–effectiveness analysis	4	5	4	4	4	5
5. Developing operational manuals for prevention programmes	6	6	6	6	5	4
6. Developing a database summarizing research to guide the general public	5	4	5	5	6	6

NA: not applicable.

^a^ Rank awarded by survey respondents in round 2 of the study to the importance of the research question for high-income and low- and middle-income countries combined. The rank was based on the mean ranking score awarded by respondents and ranges from 1 for highest to 7 for lowest, as appropriate. The number of respondents for each type of violence is given.

The ranking of broad subtypes of research questions within each step also showed marked trends across types of violence, particularly for steps 1 and 3. For step 1, research on the magnitude and distribution of violence was ranked highest for all types of violence except armed violence. For step 2, research on protective factors was ranked highest for four of the six types of violence. For step 3, research on evaluating the effectiveness of programmes that target actual violence was ranked highest for all types other than sexual violence. For step 4, participants ranked research on adapting effective programmes to new contexts highest for four of the six types of violence. Results for the cross-cutting questions are available from the corresponding author.

### Second survey

In round 2, step 3 of the public health approach (i.e. developing, implementing and evaluating interventions) was awarded the highest priority for most types of violence for all country income levels combined (Table 2) and the second highest priority for low- and middle-income countries (results for low- and middle-income countries are available from the corresponding author). We decided to focus on step 3 in round 3 because the aim of the study was to establish global research priorities for interpersonal violence prevention rather than priorities for low- and middle-income countries specifically.

The second survey involved 131 experts scoring 34 interventions and seven cross-cutting questions applicable to step 3 using five criteria (Box 2) for both high-income and low- and middle-income countries. In Table 3 (available at: http://www.who.int/bulletin/volumes/95/1/16-172965), the 34 intervention research questions are listed by their overall research priority score, which was the mean score across all five criteria expressed as a percentage. Overall scores ranged from 83.4% to 70.0%. Across all items, scores for high-income and low- and middle-income countries were similar: the mean difference was 1% (standard deviation: 1%) and the maximum difference was 4.8%, which was for “increasing access to prenatal and postnatal services in health-care settings”.

**Table 3:** Ranking of research into interventions to prevent or respond to interpersonal violence, survey of global research priorities, 2010–2013

Rank	Intervention to be researched	Mean research priority score^a^ (%)			Mean intra-class correlation^b^	Type of intervention^c^	Type of violence	Risk or protective factor targeted by intervention
		All countries	High-income countries	Low- and middle-income countries				
1	Parent–child programmes that include parenting education, child education and social support	83.4	83.8	83.0	0.85	Selective	Child maltreatment	Parenting
2	Laws to regulate and restrict civilian access to and use of small arms or firearms in public and in homes	83.3	83.0	83.6	0.84	Universal	Armed violence	Firearms
3	School-based programmes to address dating violence, gender norms and attitudes	81.6	81.2	82.1	0.85	Universal	Intimate partner violence	Norms or laws or both
4	Education about violence and abuse for health-care professionals and social workers	81.4	82.9	79.9	0.88	Universal	All types of violence	Norms or laws or both
5	Home visit programmes to improve child health and parental caregiving	80.9	81.6	80.2	0.89	Selective	Child maltreatment	Parenting
6	Life-skills interventions for all ages that address relationship and communication skills to prevent gender-based violence	80.7	81.3	80.1	0.87	Universal	Intimate partner violence, sexual violence	Norms or laws or both
7	Increasing access to prenatal and postnatal services in health-care settings	80.1	82.5	77.7	0.91	Universal	Child maltreatment	Parenting
8	Programmes to assist parents or caregivers who are experiencing family violence	80.0	79.2	80.9	0.88	Indicated	Child maltreatment, intimate partner violence	Parenting
9	Programmes to reduce physical and humiliating punishment in schools	80.0	81.2	78.7	0.89	Universal	Child maltreatment	Norms or laws or both
10	Programmes that counter social and cultural norms supportive of violence	79.7	79.8	79.6	0.90	Universal	All types of violence	Norms or laws or both
11	Anti-bullying programmes	78.8	79.4	78.2	0.89	Universal	Youth violence	Norms or laws or both
12	Social development programmes for children and adolescents that build emotional and behavioural competencies	78.7	78.2	79.3	0.85	Universal	Youth violence	ND
13	Advocacy and financial and social support programmes for victims of violence that provide advice, counselling or safety planning	78.2	78.1	78.3	0.90	Indicated	Intimate partner violence	ND
14	Training for children and adolescents on recognizing potentially abusive situations	78.1	78.4	77.7	0.90	Universal	Child maltreatment	ND
15	Programmes to prevent the early development of violent behaviour in children	77.9	77.5	78.2	0.82	Selective	Youth violence	Parenting
16	Increasing the availability and quality of child-care facilities	77.7	77.9	77.5	0.91	Universal	Child maltreatment	ND
17	Identifying victims of intimate partner violence and referral to gender-informed programmes	77.5	77.9	77.2	0.86	Indicated	Intimate partner violence	ND
18	Education about violence and abuse for people working with children in informal settings	77.3	76.8	77.8	0.92	Universal	Child maltreatment	Norms or laws or both
19	Understanding the optimal balance between criminal justice and law-enforcement responses to interpersonal violence and the primary prevention of interpersonal violence	75.4	75.1	75.7	0.92	Universal	All types of violence	ND
20	Psychological interventions to treat mental health problems associated with violence	75.3	74.2	76.4	0.90	Indicated	All types of violence	ND
21	Preschool enrichment programmes that provide children with academic and social skills at an early age	75.0	75.7	75.4	0.88	Selective	Youth violence	ND
22	Specific policing strategies, such as community or problem-oriented policing, to prevent violence	74.6	75.3	74.0	0.91	Selective	Youth violence	ND
23	Creating safe routes for children on their way to and from school or other community activities	74.5	75.6	73.4	0.90	Universal	Youth violence, sexual violence	ND
24	Formal processes for the use of data on injuries due to assault derived from accident and emergency departments to reduce city violence (Cardiff Model)	74.4	74.0	74.8	0.92	Selective	Youth violence, armed violence	ND
25	Mass media campaigns to prevent violence	73.9	74.2	73.5	0.94	Universal	All types of violence	Norms or laws or both
26	Regulating sales of alcohol to lower consumption (e.g. reducing sales hours or the number of retail outlets, raising prices)	73.8	73.6	74.0	0.93	Universal	All types of violence	Alcohol
27	Monitoring and improving adherence by national governments to treaties or laws protecting human rights	73.8	75.7	71.8	0.91	Universal	All types of violence	ND
28	Providing after-school programmes to extend adult supervision	72.8	72.5	73.0	0.91	Universal	Youth violence	ND
29	Improving alcohol-drinking environments (e.g. reducing crowding, late-night transport, education to reduce binge drinking)	72.3	72.1	72.6	0.93	Universal	All types of violence	Alcohol
30	Microfinance combined with gender equity training to reduce gender-based violence	71.8	74.1	69.5	0.93	Selective	Intimate partner violence	Poverty or inequality or both
31	Specialized gang and street violence prevention strategies such as targeted deterrence and Cure Violence	71.8	70.6	73.0	0.93	Selective	Youth violence, armed violence	Norms or laws or both
32	Protection orders that prohibit the perpetrator from contacting the victim	71.4	71.5	72.4	0.94	Indicated	Intimate partner violence	Norms or laws or both
33	Mandatory reporting of suspected violence or abuse	70.5	71.5	69.6	0.93	Indicated	All types of violence except youth violence and armed violence	Norms or laws or both
34	Brief interventions and treatment for problem drinkers (e.g. cognitive behavioural therapy)	70.0	70.0	69.9	0.92	Indicated	All types of violence	Alcohol

ND: not determined.

^a^ The research priority score was the mean of scores awarded by 131 survey respondents across five criteria for research on violence prevention (Box 2). For each of the 34 questions, we calculated the mean rating and expressed it as a percentage (rather than out of 5). For example, if the mean rating across the five criteria was 3.8, we reported 76% (3.8/5).

^b^ The intra-class correlation indicates the level of agreement across survey respondents. A correlation of 0.75 or above was considered excellent.

^c^ Universal interventions are directed at the whole population, selective interventions target high-risk subpopulations and indicated interventions target populations that have already been exposed to violence.

We examined three characteristics of the 34 interventions: (i) whether they were universal (i.e. directed at the whole population regardless of risk), selective (i.e. targeted at higher-risk subpopulations) or indicated (i.e. targeted populations that had already been exposed to violence); (ii) the type of violence they primarily addressed; and (iii) the risk factor they principally aimed to reduce. Universal and selective interventions had similar mean research priority scores (77.6% and 76.2%, respectively); the score for indicated interventions was 74.7%. Interventions that addressed child maltreatment had the highest mean score (79.7%), followed by those that addressed sexual violence (77.6%), intimate partner violence (77.3%), armed violence (76.5%), youth violence (75.4%) and all types of violence (75.1%). The single intervention that addressed the use of firearms as a risk factor had the highest mean score (83.3%), followed by those that addressed parenting (80.5%), social norms or laws (77.0%), alcohol (72.0%) and poverty or inequality (71.8%).

In response to a question about prioritizing research according to the type of violence, out of the 131 respondents, 58% (76) regarded it as possible, 28% (37) regarded it as not possible and 14% (18) expressed no view. There was no association between the respondent’s area of expertise and their response. The mean priority ranking for the different types of violence, from 1 for highest to 6 for lowest, was child maltreatment (2.05), intimate partner violence (3.22), youth violence (3.46), armed violence (3.96), sexual violence (4.07) and elder abuse (4.43). There was no association between the respondent’s area of expertise and the type of violence assigned the highest priority, except for child maltreatment, where Spearman’s rank correlation coefficient was 0.54 (*P* < 0.0001).

## Discussion

Globally, our priority-setting exercise found that research on the development, implementation and evaluation of interventions – step 3 of the public health approach to violence prevention – was ranked as having the highest priority. Research on identifying risk and protective factors and the causes and correlates of violence (step 2) was ranked second highest, though somewhat less consistently across different types of violence. Research describing the nature, magnitude, distribution and consequences of violence (step 1) was ranked third highest for most types of violence, with the notable exception of elder abuse, for which it ranked highest. The most consistent finding, however, was that scaling up interventions and evaluating their cost–effectiveness (step 4) ranked lowest across all types of violence.

One explanation for our main findings is that respondents considered it premature to scale up interventions (step 4) before there is sufficient evidence of an intervention’s effectiveness (step 3). Such an interpretation is consistent with the findings of recent systematic reviews of interventions to prevent and respond to different forms of violence, which suggest that the evidence base remains thin and substantial investment in research is required.^20^^–^^23^ Another possible interpretation is that respondents thought countries may lack the political will to scale up violence prevention interventions they view as being too costly or may lack the capacity to scale them up.^24^^,^^25^ Although our findings converge with those of similar priority-setting exercises that focused on child maltreatment and intimate partner violence in high-income countries^16^ and on adolescent sexual and reproductive health, including gender-based violence, in low- and middle-income countries,^18^ they stand in stark contrast to recent calls to scale up violence prevention interventions.^26^

Our finding that research on identifying risk and protective factors and the causes and correlates of violence (step 2) had the second highest priority overall, and the highest priority in low- and middle-income countries, concurs with recent reviews that concluded that the evidence base in this area is still limited, particularly on the causal status of risk factors and their relative importance.^27^^,^^28^ Given the gaps in knowledge about the prevalence of fatal and nonfatal violence existing in many countries,^1^ it is surprising that step 1, which includes describing the magnitude and distribution of violence, was ranked third for most types of violence. Perhaps respondents considered the gaps in research on other steps as comparatively greater and of more pressing concern. Also, it is possible that respondents were based in countries for which adequate knowledge of the magnitude of violence was available and they lacked a more global perspective.

Two noteworthy findings emerged in round 3 on ranking the 34 more detailed, intervention research questions. First, highest ranked questions were interventions that addressed violence against children and violence against women, both sexual and intimate partner violence. This may reflect the prominence of these types of violence on international agendas and acknowledges the importance of violence against children as a risk factor for involvement in other forms of violence, such as youth violence and intimate partner violence, throughout those children’s lives.^29^^,^^30^ Second, among interventions that targeted risk factors, those that addressed firearms or parenting were ranked highest, whereas those that addressed alcohol or poverty and social inequality were ranked lowest. However, in the absence of detailed, well-supported evidence on the relative importance of different risk factors for most types of violence and given the lack of consensus on other risk factors, such as the relative importance of poverty and social inequality as a risk factor for homicide,^31^ these rankings may primarily reflect respondents’ perceptions.

Our priority-setting exercise has several strengths. First, the number of experts who participated in the surveys and the number of countries, sectors and organizations they represented (Table 1) are as large or larger than most similar global research priority-setting exercises.^13^^,^^15^^,^^16^ Second, the hybrid Delphi–Child Health and Nutrition Research Initiative method allowed us to identify priorities among and within the steps of the public health approach in the context of a complex field.

The study has several limitations. First, the response rate in round 2 was only 40% after follow-up reminders and there was an attrition rate of 53% between rounds 2 and 3. However, these response and attrition rates are in line with those of similar priority-setting exercises.^12^^,^^15^ A comparison of the available demographic characteristics of respondents and nonrespondents indicated they were similar but it is possible they differed on variables we were unable to assess. Second, the extent to which respondents were representative of the global community of violence prevention experts is unknown. Nevertheless, the WHO and Violence Prevention Alliance networks we used to identify potential respondents are probably among the most extensive in the world. Third, use of the public health approach to organize research priorities may have dissuaded those unfamiliar with this approach from completing the surveys. However, the interventions respondents were asked to prioritize in round 3 were not specific to the public health approach and included interventions with which most experts were likely to have been familiar. Fourth, the length of the surveys and the interval between rounds 2 and 3 of almost 1 year may have discouraged some potential respondents. Fifth, it is possible that the decision taken in round 3 to focus on more detailed research priorities related to the step of the public health approach ranked highest in round 2, namely step 3, may have precluded the emergence of more detailed research priorities related to another step of the public health approach. Finally, this paper focused on the global results of this research priority-setting exercise; more finely grained analyses by region, country-income level and individual country will be published in the future.

This priority-setting exercise on global research into violence prevention showed that scaling up violence prevention interventions was consistently awarded the lowest priority, whereas developing, implementing and evaluating interventions was awarded the highest. It appears that a massive investment in outcome evaluations, which matches the global burden of violence, is required before the field is ready to scale up preventive measures. The hope is that, within a decade, enough evidence will have accumulated to start scaling up interventions that will help achieve the ambitious SDG targets of altogether eliminating some forms of violence from the world and substantially reducing others by 2030.
