# Corrigendum

**DOI:** 10.2471/BLT.17.110217

**Published:** 2017-02-01

In Volume 95, Issue 1, January 2016, page 45 should have read: Nevertheless, the WHO and Violence Prevention Alliance networks we used to identify potential respondents are probably among the most extensive in the world. Third, use of the public health approach to organize research priorities may have dissuaded those unfamiliar with this approach from completing the surveys. However, the interventions respondents were asked to prioritize in round 3 were not specific to the public health approach and included interventions with which most experts were likely to have been familiar. Fourth, the length of the surveys and the interval between rounds 2 and 3 of almost 1 year may have discouraged some potential respondents. Fifth, it is possible that the decision taken in round 3 to focus on more detailed research priorities related to the step of the public health approach ranked highest in round 2, namely step 3, may have precluded the emergence of more detailed research priorities related to another step of the public health approach. Finally, this paper focused on the global results of this research priority-setting exercise; more finely grained analyses by region, country-income level and individual country will be published in the future.

This priority-setting exercise on global research into violence prevention showed that scaling up violence prevention interventions was consistently awarded the lowest priority, whereas developing, implementing and evaluating interventions was awarded the highest. It appears that a massive investment in outcome evaluations, which matches the global burden of violence, is required before the field is ready to scale up preventive measures. The hope is that, within a decade, enough evidence will have accumulated to start scaling up interventions that will help achieve the ambitious SDG targets of altogether eliminating some forms of violence from the world and substantially reducing others by 2030: Mikton CR, Tanaka M, Tomlinson M, Streiner DL, Tonmyr L, Lee BX et al. Global research priorities for interpersonal violence prevention: a modified Delphi study. Bull World Health Organ. 2017;95(1):37–49. http://dx.doi.org/10.2471/BLT.16.172965.

